# Clinical utility of fecal Sanger sequencing and PCR alongside gastric biopsy E-test for *Helicobacter pylori* resistance to clarithromycin and quinolone

**DOI:** 10.1128/spectrum.04062-25

**Published:** 2026-04-14

**Authors:** Juan Xu, Linjie Peng, Haitao Hu, Yan Zhao, Wang Li, Hailin Peng, Chenglin Zhou

**Affiliations:** 1 Clinical Laboratory Center, The Affiliated Taizhou People’s Hospital of Nanjing Medical University 372209 , Taizhou, China; 2 Taizhou School of Clinical Medicine, Nanjing Medical University 372209, Taizhou, China; MultiCare Health System, Tacoma, Washington, USA

**Keywords:** *Helicobacter pylori*, clarithromycin resistance, quinolone resistance, *23S rRNA*, *gyrA*, resistant phenotype, resistant genotype, E-test, Sanger sequencing, PCR

## Abstract

To guide *Helicobacter pylori* eradication, we determined the prevalence and predictability of clarithromycin and quinolone resistance in 277 consecutive culture-positive patients who underwent gastroscopy. Gastric biopsy isolates were tested phenotypically by E-test, while paired fecal DNA was examined genotypically by Sanger sequencing and a PCR melting curve method for the *23S rRNA* (clarithromycin) and *gyrA* (quinolone) mutation hotspots. Phenotypic resistance was detected in 172/233 (73.8%) and 157/232 (67.7%) isolates to clarithromycin and quinolones, respectively. The predominant mutations were A2143G in *23S rRNA* and N87K_C261A in *gyrA*. Genotypic resistance rates were 70.1% (162/231) by sequencing and 69.1% (168/243) by PCR for clarithromycin, and 65.2% (133/204) and 64.5% (151/234) for quinolones. Resistance increased significantly with patient age (*P* < 0.01 for both drugs). Genotype-phenotype concordance was high: 90.1% (κ = 0.751) and 93.3% (κ = 0.849) for Sanger sequencing, and 88.2% (κ = 0.706) and 92.6% (κ = 0.834) for PCR, for clarithromycin and quinolones, respectively. These data demonstrate an alarming burden of primary *H. pylori* resistance that rises with age. Fecal PCR-based genotyping reliably anticipates the *in vitro* susceptibility pattern and can therefore be used to tailor empirical therapy without the need for invasive culture.

**IMPORTANCE:**

In the studied region, primary resistance to clarithromycin and quinolones in *Helicobacter pylori* exceeds 65% and rises sharply with age. Non-invasive fecal PCR or Sanger genotyping of *23S rRNA* and *gyrA* mutations showed ≥85% agreement with invasive E-test results, enabling age-stratified, resistance-guided eradication therapy without endoscopy or culture.

## INTRODUCTION

*Helicobacter pylori* infection is a major cause of chronic gastritis (1, 2) and peptic ulcers (3). In severe cases, it can lead to gastric cancer and mucosa-associated lymphoid tissue (MALT) lymphoma (4, 5). Eradication of *H. pylori* is a critical measure to reduce the risk of these associated diseases (3, 6). Triple therapies based on clarithromycin and levofloxacin have been widely used as first-line treatments (7). However, in recent years, the resistance rates of *H. pylori* to clarithromycin and levofloxacin have reached alarming levels worldwide (8, 9). Resistance has reduced the success rates of clarithromycin- and levofloxacin-based triple therapies by approximately 50% (10) and 20%–40% (11), respectively. In many settings, this trend has been driven at least in part by inappropriate or unregulated use of antibiotics, which has accelerated the emergence and spread of resistant *H. pylori* strains (12). Current guidelines no longer recommend traditional empirical therapies in regions with high clarithromycin resistance (13). Although studies on levofloxacin resistance are less extensive, the increasing number of clinical resistance cases and empirical treatment failures have led researchers to suggest that *H. pylori* treatment should be guided by antimicrobial susceptibility testing and resistance gene detection (14).

Although *H. pylori* eradication is a significant public health initiative in China, data on regional resistance rates to clarithromycin and levofloxacin are still lacking. Consequently, the applicability of empirical therapies based on these antibiotics in clinical practice lacks theoretical support. Therefore, analyzing resistance to clarithromycin and levofloxacin in the population is not only a valuable addition to the existing research but also a key foundation for developing effective *H. pylori* eradication strategies.

In recent years, advances have been made in *H. pylori* diagnosis, but phenotypic antimicrobial susceptibility testing based on bacterial culture remains the most commonly used method for determining antibiotic resistance (15). However, isolating and culturing *H. pylori* from gastric biopsy samples is an invasive procedure and involves several technical challenges, such as variability in clinical specimen quality, differences in microbial community distribution, and inappropriate transport conditions that can affect culture outcomes (16). Moreover, culture-based methods require highly trained laboratory personnel and can take up to 14 days to yield results. Consensus guidelines recommend non-invasive tests such as stool antigen assays or urea breath tests, but these methods have not yet achieved satisfactory clinical performance (17, 18). Therefore, exploring rapid and accurate molecular methods to assess *H. pylori* resistance is of great clinical value, especially for diagnosing clarithromycin and quinolone resistance.

Studies have shown that the primary mechanism of clarithromycin resistance involves point mutations in the peptidyl transferase region of the *23S rRNA* gene (A2143G, A2142G, and A2142C) (19, 20). As the binding target of clarithromycin, the *23S rRNA* peptidyl transferase center in *H. pylori* is a crucial component of the ribosomal subunit. Mutations at positions A2142 and A2143 induce conformational changes that significantly reduce ribosomal-binding affinity for clarithromycin, leading to antimicrobial resistance (21). Notably, *H. pylori* harbors two *23S rRNA* operons (rrnA and rrnB) that are highly conserved and share identical sequences (22). Genetic analyses have revealed that clinical *H. pylori* strains typically exhibit homozygous mutations at these loci, whereas heterozygous mutations remain exceptionally rare and have been reported only in *in vitro* site-directed mutagenesis experiments (23). The mechanism of fluoroquinolone action involves inhibition of DNA gyrase and topoisomerase, enzymes critical for controlling and modifying the topological state of DNA in bacterial cells (24). However, unlike many other bacterial species, *H. pylori* lacks the gene encoding topoisomerase IV, an important fluoroquinolone target (25). Consequently, resistance to quinolones is predominantly associated with point mutations at codon positions 87 and 91 of the *gyrA* gene (26). Although non-invasive detection of resistance genes using stool samples has been proposed, clinical data on such methods remain limited. The sensitivity of these approaches has not been fully validated for clinical use. Additionally, due to the high sequence variability of the *gyrA* gene, designing effective PCR-based detection methods remains a complex challenge. Research on PCR-based detection of quinolone resistance from stool samples is particularly scarce (27).

Our team previously participated in developing a real-time PCR assay to detect clarithromycin and quinolone resistance genotypes directly from stool samples. In this study, we enrolled *H. pylori*-infected individuals and used multiple approaches—including conventional E-test antimicrobial susceptibility testing, Sanger sequencing, and our self-developed PCR method—to assess resistance to clarithromycin and quinolones. We analyzed the correlation between resistance and clinical parameters and compared the concordance between phenotypic and genotypic resistance. The aim is to provide a more comprehensive theoretical basis for the rational selection of treatment regimens in *H. pylori*-infected populations.

## MATERIALS AND METHODS

### Study population

From May to December 2023, 277 consecutive subjects who underwent upper gastrointestinal endoscopy at Taizhou People’s Hospital and were diagnosed with *H. pylori* infection were enrolled. There were 148 males (53.4%) and 129 females (46.6%), with a mean age of 50.5 ± 12.0 years. Among them, 219 (79.1%) were treatment-naïve, and 58 (20.9%) had previously received eradication therapy.

### Sample collection and storage

During endoscopy, one gastric mucosal biopsy was immediately snap-frozen in liquid nitrogen for bacterial culture and phenotypic susceptibility testing. A synchronous stool specimen was collected into a commercial preservative tube (Taizhou Kangwei Biotech, 20190177) and stored at −20°C ± 5°C until nucleic acid extraction for PCR-based resistance genotyping and Sanger sequencing.

### Procedures

#### Instruments and reagents

E-test strips for clarithromycin and levofloxacin were used according to the manufacturer’s instructions (Wenzhou Kangtai, 20152400299). MIC breakpoints were ≤0.25 mg/L (susceptible) and >0.25 mg/L (resistant) for clarithromycin, and ≤1 mg/L (susceptible) and >1 mg/L (resistant) for levofloxacin according to EUCAST v13.0 guidelines.

Genomic DNA was extracted from 200 mg of stool using the Kangwei Century DNA extraction kit (Jiangsu Taizhou medical device record no. 20200970). Real-time PCR was performed on an ABI 7500 platform. Primers used were as follows: for *23S rRNA*, 23ScexuF (5′-ATGGGAGCTGTCTCAACCAGAG-3′) and 23ScexuR (5′-CTCCATAAGAGCCAAAGCCCTTACT-3′); for *gyrA*, gyrAcexuF (5′-GATCGTGGGTGATGTGATTGGTA-3′) and gyrAcexuR (5′-GCCTTAGTCATTCTGGCTTCAGTG-3′).

#### Culture and phenotypic susceptibility testing

Biopsy tissue was homogenized under sterile conditions and suspended in brain-heart infusion broth. Twenty microliters of the suspension was plated onto *H. pylori*-selective medium containing 5% horse blood, clarithromycin (0.064–256 mg/L; Wenzhou Kangtai, WE0410S1), and levofloxacin (0.008–32 mg/L; Wenzhou Kangtai, WE080251). Plates were incubated at 37°C under microaerophilic conditions (85% N₂, 10% CO₂, and 5% O₂) in a HealForce HF100 tri-gas incubator (Shanghai LiShen Scientific Instrument) for 4–7 days. Growth was checked every 48 h; isolates with insufficient growth were recorded as missing values (see Tables S1 and S2). MICs were read at 72 h with the E-test strip according to EUCAST v13.0 guidelines.

#### Sanger sequencing

PCR was carried out in a 50 µL volume containing 12.5 µL Fast Probe Mixture, 1 µL each primer (10 µM), 20 µL template DNA, and 15.5 µL nuclease-free water. Cycling conditions were as follows: 95°C 2 min; 45 cycles of 95°C 15 s, 60°C 1 min; final extension 72°C 5 min. Amplicons were purified and bidirectionally sequenced by Sangon Health Technology (Shanghai). Chromatograms were analyzed with Chromas 2.6; mutations A2142G/C and A2143G in *23S rRNA* and codons 87/91 of *gyrA* were regarded as resistance markers.

### Statistical analysis

Analyses were performed with SPSS 22.0. Categorical variables were compared by χ² test; agreement between phenotypic and genotypic results was assessed with Cohen’s κ coefficient. The Kruskal-Wallis test was used for multiple-group comparisons. A two-sided *P* value< 0.05 was considered statistically significant.

## RESULTS

### Analysis of clarithromycin and quinolone resistance phenotypes in *H. pylori*-infected individuals

**Fig 1:**
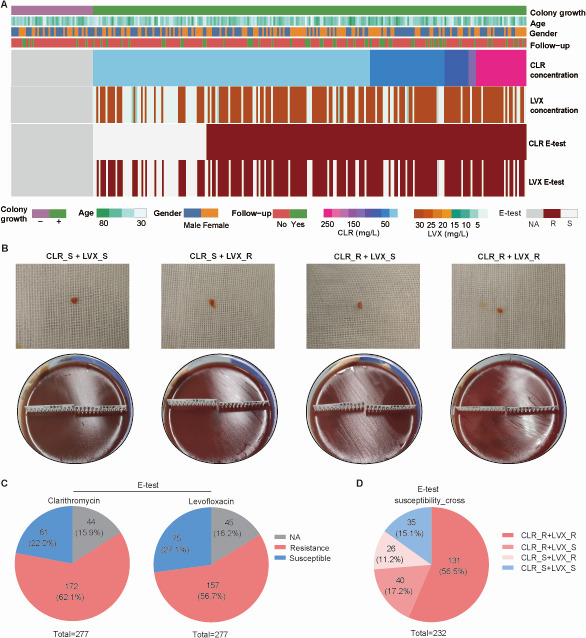
E-test-based phenotypic analysis of *H. pylori* clarithromycin and levofloxacin resistance. (**A**) Heatmap summarizing baseline clinical characteristics and phenotypic susceptibility to clarithromycin (CLR) and levofloxacin (LVX) of the enrolled subjects (*n* = 277). (**B**) Representative endoscopic gastric mucosa images and E-test strips for each resistance category. (**C**) Pie charts showing the proportions of CLR- and LVX-resistant, susceptible, or missing-phenotype isolates. (**D**) Pie charts depicting the distribution of CLR and LVX resistance phenotypes. CLR, Clarithromycin; LVX, Levofloxacin; CLR_R, Clarithromycin Resistance; CLR_S, Clarithromycin Susceptible; LVX_R, Levofloxacin Resistance; LVX_S, Levofloxacin Susceptible. Follow-up No, First visit; Follow-up Yes, Follow-up visit.

In all, 277 subjects were enrolled, and gastric mucosal tissues were collected for *in vitro H. pylori* culture using the E-test. Clinical parameters, including age, gender, and primary diagnosis/retreatment status, were collected simultaneously. Among the enrolled subjects, 44 cases were *H. pylori* culture negative in the clarithromycin E-test (15.9%, 44/277) and 45 cases were *H. pylori* culture negative in the quinolone E-test (16.2%, 45/277). Consequently, 233 cases (84.1%, 233/277) were included in the clarithromycin resistance analysis and 232 cases (83.8%, 232/277) in the quinolone resistance analysis (Fig. 1A and B). For clarithromycin resistance phenotype analysis, among the 233 enrolled subjects, 172 were clarithromycin resistant (73.8%, 172/233) and 61 were clarithromycin sensitive (26.2%, 61/233). For quinolone resistance phenotype analysis, among 232 enrolled subjects, 157 were levofloxacin resistant (67.7%, 157/232) and 75 were levofloxacin sensitive (32.3%, 75/232) (Fig. 1C). Dual resistance to both clarithromycin and quinolone was observed in 131 cases (56.5%, 131/232). Clarithromycin-resistant but quinolone-sensitive cases numbered 40 (17.2%, 40/232), clarithromycin-sensitive but quinolone-resistant cases were 26 (11.2%, 26/232), and cases sensitive to both were 35 (15.1%, 35/232) (Fig. 1D).

### Correlation analysis between clarithromycin/quinolone resistance phenotypes and clinical parameters

**Fig 2:**
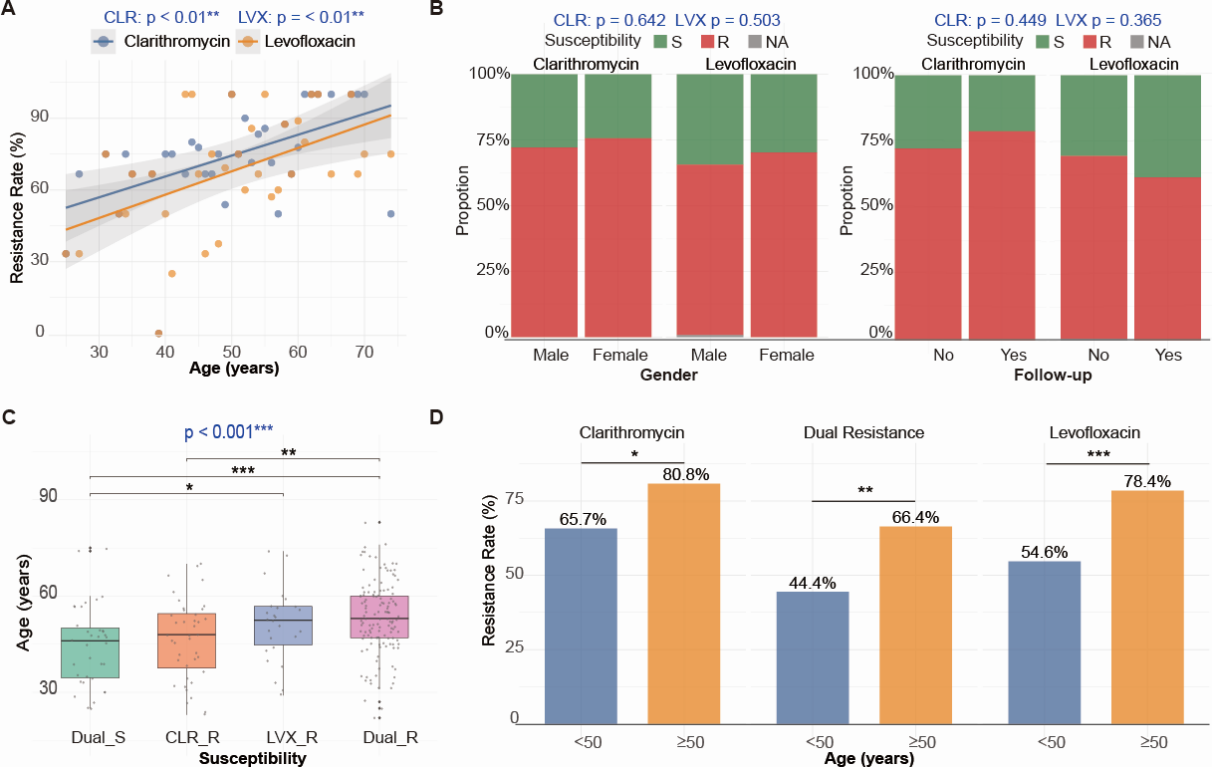
Association between CLR/LVX resistance phenotypes and clinical parameters. (**A**) Scatter plots evaluating the correlation between CLR or LVX resistance phenotype and patient age. (**B**) Stacked bar charts illustrating resistance phenotype distribution by sex and follow-up status. (**C**) Bar chart comparing age distribution across phenotype groups. (**D**) Bar chart comparing the proportions of single-drug and dual resistance among different age strata. ^*^P < 0.05, ^**^P < 0.01, ^***^P < 0.001.

When grouped by gender, no significant differences were found in the resistance rates of clarithromycin (χ² = 0.378, *P* = 0.539) or quinolone (χ² = 0.657, *P* = 0.418) phenotypes between males and females. Similarly, no significant differences were observed in the resistance rates of clarithromycin (χ² = 0.875, *P* = 0.350) or quinolone (χ² = 1.153, *P* = 0.283) phenotypes between primary diagnosis and retreatment groups. However, individuals aged >50 years showed significantly higher resistance rates for both clarithromycin (χ² = 6.799, *P* = 0.009) and quinolone (χ² = 14.257, *P* = 0.000) phenotypes compared to those ≤50 years (Table 1). Correlation analysis revealed that resistance rates for clarithromycin and quinolone increased significantly with age (*P* < 0.01) (Fig. 2A), but showed no correlation with gender or primary/retreatment status (*P* > 0.05) (Fig. 2B). Based on resistance phenotypes, subjects were divided into four groups: dual sensitive (Dual_S), clarithromycin-resistant only (CLR_R), quinolone-resistant only (LVX_R), and dual resistant (Dual_R). Comparison of age differences among these groups showed that the Dual_R group was significantly older than the Dual_S and CLR_R groups. The LVX_R group was also significantly older than the Dual_S group (*P* < 0.05) (Fig. 2C). Grouping by median age revealed that the resistance rates in the ≥50-year group were significantly higher than those in the <50-year group (*P* < 0.05) (Fig. 2D).

**TABLE 1:** Association between clarithromycin/levofloxacin resistance phenotypes and clinical parameters^*a*^

Clinical parameters	Clarithromycin				Levofloxacin			
	Resistant	Susceptible	*χ*²	*P* value	Resistant	Susceptible	*χ*²	*P* value
Sex								
Male	88	34	0.378	0.539	79	42	0.657	0.418
Female	84	27			78	33		
Age (years)								
<50	71	37	6.799	0.009**	59	48	14.257	0.000***
≥50	101	24			98	27		
Number of visits								
First visit	131	50	0.875	0.350	125	55	1.153	0.283
Follow-up visit	41	11			32	20		

^**^ *P* < 0.01, ^***^P < 0.001.

### Sanger sequencing analysis of clarithromycin and quinolone resistance genotypes

**Fig 3:**
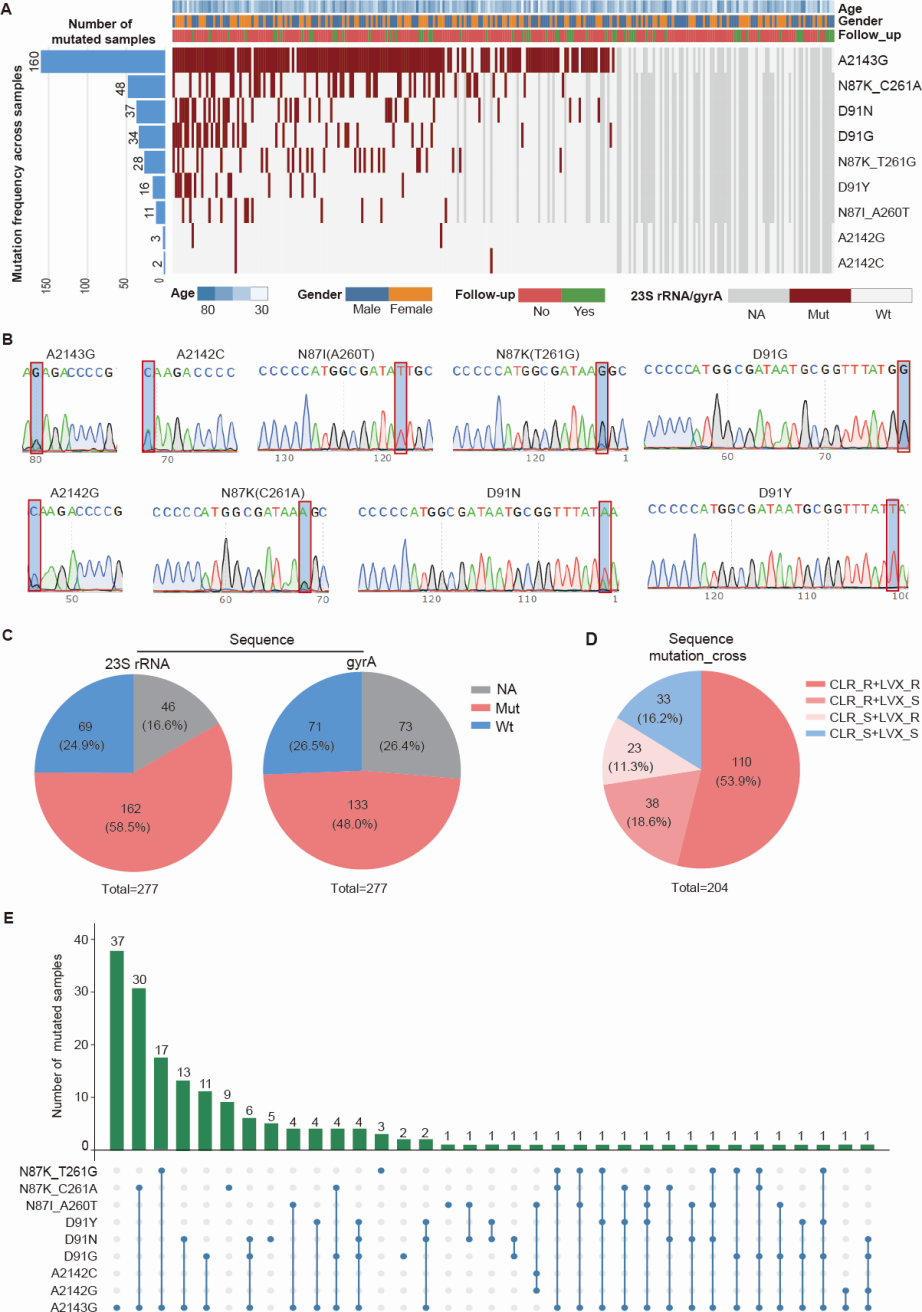
Sanger-sequencing analysis of *H. pylori* genotypic resistance to CLR and LVX. (**A**) Heatmap of baseline clinical data alongside *23S rRNA* and *gyrA* mutation status (*n* = 277). (**B**) Representative Sanger sequencing chromatograms for key *23S rRNA* and *gyrA* mutation sites. (**C**) Pie charts indicating the percentages of genotypic resistance to CLR and LVX. (**D**) Pie charts displaying the distribution of *23S rRNA* and *gyrA* resistance-associated mutations. (**E**) UpSet plot summarizing the co-occurrence patterns of individual *23S rRNA* and *gyrA* mutation sites. Follow-up No, First visit; Follow-up Yes, Follow-up visit.

Sanger sequencing analyzed three loci (A2142C, A2142G, and A2143G) in the *23S rRNA* gene and six loci (D91N, D91G, D91Y, N87K_C261A, N87K_T261G, and N87I_A260T) in the *gyrA* gene. Mutation at the A2143G locus was the most common, detected in 160 subjects (Fig. 3A and B). For the clarithromycin resistance gene *23S rRNA*, 46 cases yielded invalid results. Among 231 subjects included in the analysis, 162 (70.1%, 162/231) had mutant-type *23S rRNA*, and 69 (29.9%, 69/231) had wild type. For the quinolone resistance gene *gyrA*, 73 cases yielded invalid results. Among 204 subjects included in the analysis, 133 (65.2%, 133/204) had mutant-type *gyrA*, and 71 (34.8%, 71/204) had wild type (Fig. 3C). Both *23S rRNA* and *gyrA* mutations were found in 110 cases (53.9%, 110/204). *23S rRNA* mutation with *gyrA* wild type occurred in 38 cases (18.6%, 38/204), *23S rRNA* wild type with *gyrA* mutation in 23 cases (11.3%, 23/204), and both genes wild type in 33 cases (16.2%, 33/204) (Fig. 3D). Further analysis revealed that 37 subjects (18.1%, 37/204) had a single A2143G mutation, 30 (14.7%, 30/204) had dual-site mutations (A2143G and N87K_C261A), 6 (2.9%, 6/204) had triple-site mutations (D91N, D91G, and A2143G), and 4 (2.0%, 4/204) had quadruple-site mutations (A2143G, D91N, D91G, D91Y) (Fig. 3E).

### Correlation analysis between first-generation sequencing results for resistance genotypes and clinical parameters

**Fig 4:**
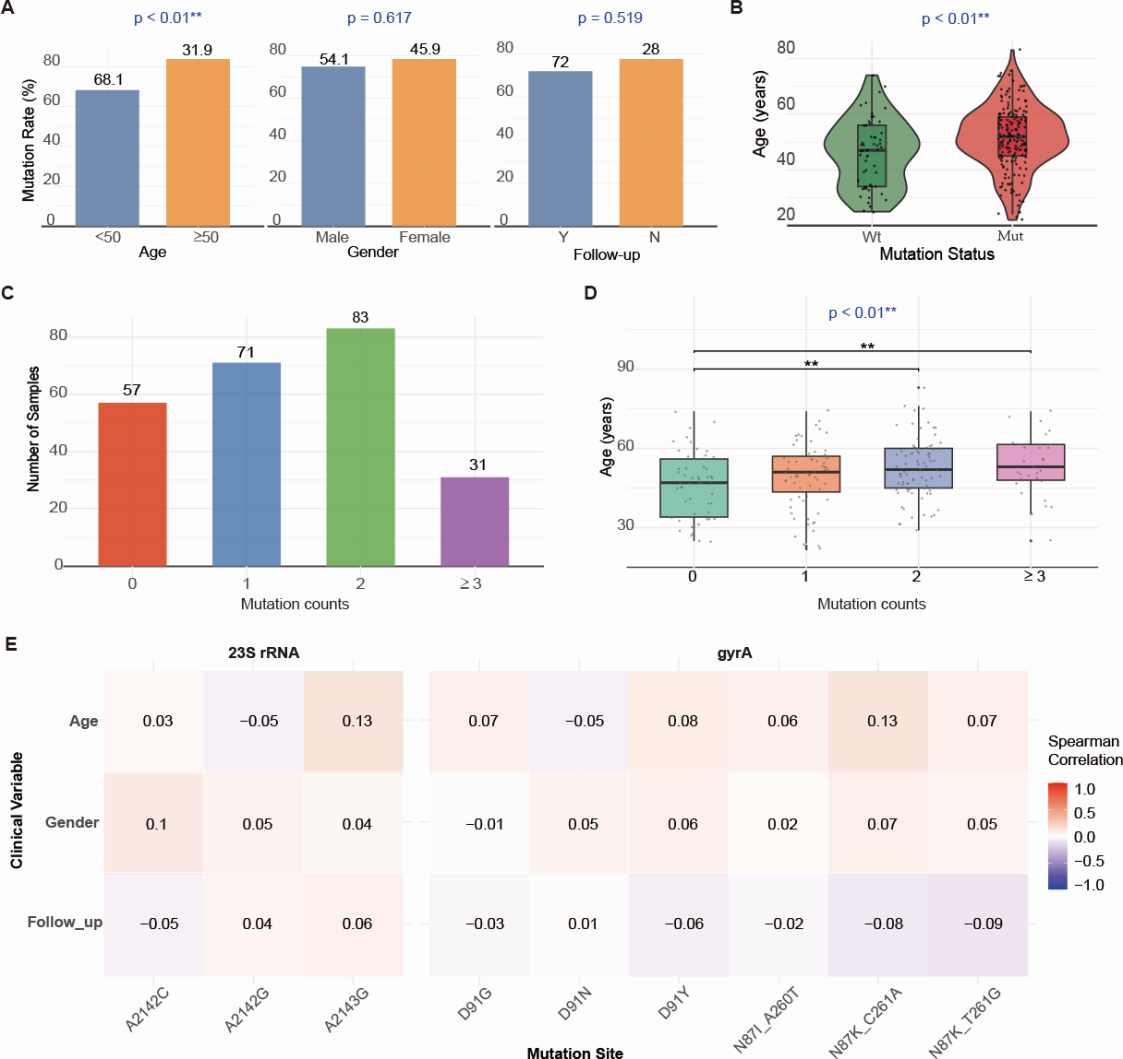
Correlation between Sanger-detected resistance mutations and clinical parameters. (**A**) Bar charts relating *23S rRNA* and *gyrA* mutation status to sex, age group, and follow-up status. (**B**) Violin plots comparing age distributions between mutated and wild-type groups for *23S rRNA* and *gyrA*. (**C**) Bar chart showing the frequency of isolates carrying different numbers of mutation sites. (**D**) Box plots comparing age differences among groups stratified by mutation-site count. (**E**) Heatmap analysis of the correlation between *23S rRNA* and *gyrA* gene mutation sites and clinical parameters. Follow-up No, First visit; Follow-up Yes, Follow-up visit. ^**^P < 0.01.

First-generation sequencing results (Table 2) showed no significant differences in clarithromycin (χ² = 0.784, *P* = 0.376) or quinolone (χ² = 1.661, *P* = 0.197) genotype resistance rates between males and females. Similarly, no significant differences were found between primary diagnosis and retreatment groups for clarithromycin (χ² = 0.615, *P* = 0.433) or quinolone (χ² = 1.512, *P* = 0.219) genotype resistance rates. Compared to the <50-year group, the ≥50-year group showed no significant change in clarithromycin genotype resistance rate (χ² = 3.031, *P* = 0.082), but had a significantly higher quinolone genotype resistance rate (χ² = 5.557, *P* = 0.018). Group analysis based on clinical parameters revealed that mutation rates for *23S rRNA* and *gyrA* genes were significantly higher in the older age group (*P* < 0.01). No significant differences were observed between males and females (*P* > 0.05) or between primary diagnosis and retreatment subjects (*P* > 0.05) (Fig. 4A). Statistical analysis showed that the age of subjects with *23S rRNA* or *gyrA* mutations was significantly higher than that of subjects with the wild-type genes (*P* < 0.01) (Fig. 4B). Furthermore, subjects with more than two mutation sites were significantly older than those with the wild type (*P* < 0.01) (Fig. 4C and D). Correlation analysis found no significant association between specific mutation sites and clinical parameters (*P* > 0.05) (Fig. 4E).

**TABLE 2:** First-generation sequencing analysis of resistance genotypes vs. clinical parameters^*a*^

Clinical parameters	*23S rRNA*				*gyrA*			
	Mut	Wt	*χ*²	*P* value	Mut	Wt	*χ*²	*P* value
Sex								
Male	86	41	0.784	0.376	68	43	1.661	0.197
Female	76	28			65	28		
Age (years)								
<50	69	38	3.031	0.082	52	40	5.557	0.018*
≥50	93	31			81	31		
Number of visits								
First visit	129	58	0.615	0.433	109	53	1.512	0.219
Follow-up visit	33	11			24	18		

^*^ *P* < 0.05. Mut, mutation; Wt, wild type.

### Fluorescence quantitative PCR analysis of clarithromycin and quinolone resistance genotypes

**Fig 5:**
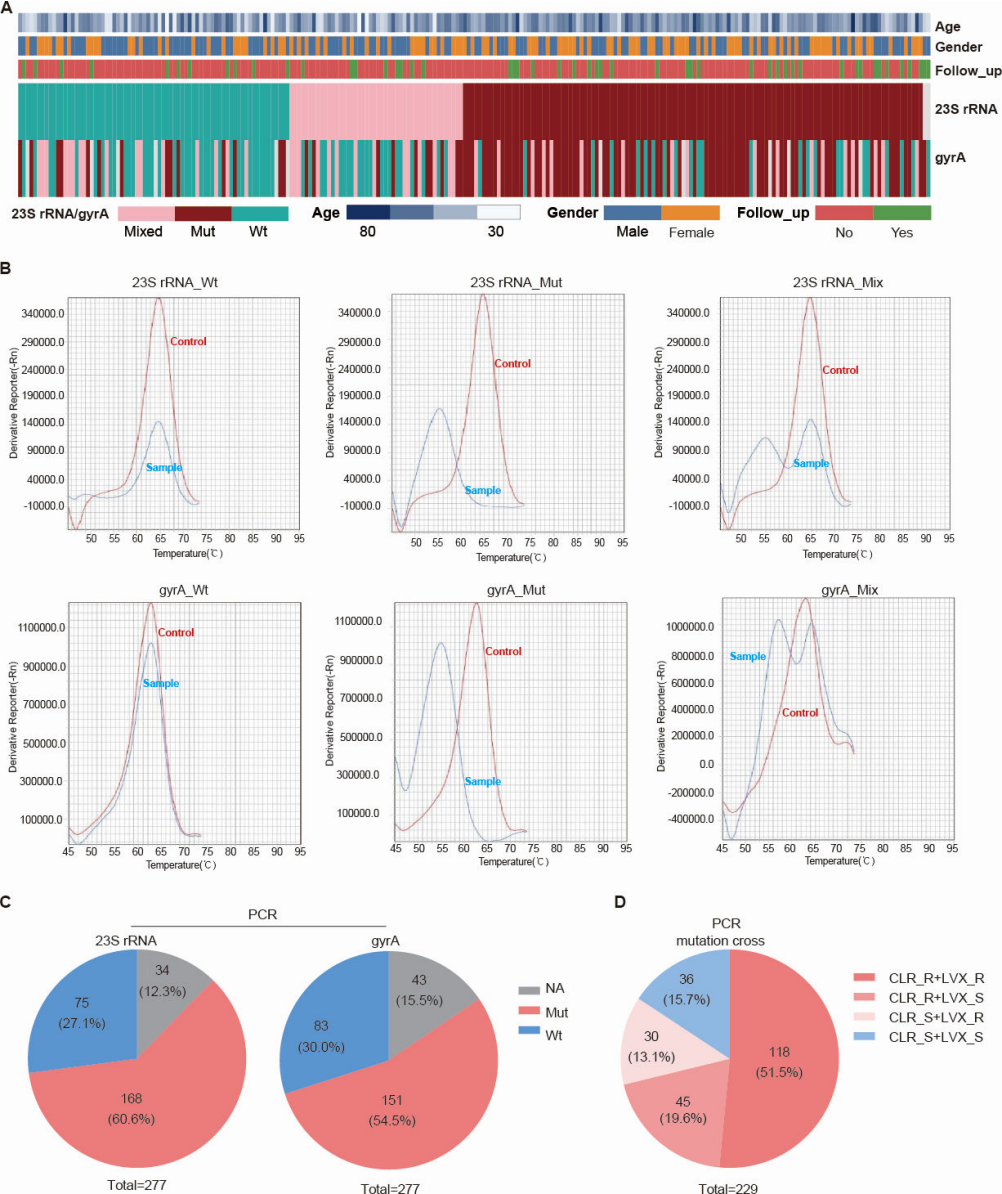
PCR-based detection of CLR and LVX resistance genotypes. (**A**) Heatmap of clinical baseline characteristics together with *23S rRNA* and *gyrA* mutation results obtained by PCR (*n* = 277). (**B**) Representative high-resolution melting (HRM) curves for key *23S rRNA* and *gyrA* mutations. (**C**) Pie charts presenting the proportions of genotypic resistance to CLR and LVX. (**D**) Pie charts illustrating the distribution of *23S rRNA* and *gyrA* resistance mutations. Follow-up No, First visit; Follow-up Yes, Follow-up visit.

Based on melting curve analysis, genotypes for *23S rRNA* and *gyrA* included wild type, mutant type, and mixed type (Fig. 5A and B). For the *23S rRNA* gene, wild type, mutant type, and mixed type were found in 75 (27.1%, 75/277), 122 (44.0%, 122/277), and 46 (16.6%, 46/277) cases, respectively. For the *gyrA* gene, wild type, mutant type, and mixed type were found in 83 (30.0%, 83/277), 101 (36.5%, 101/277), and 50 (18.0%, 50/277) cases, respectively. Fluorescence quantitative PCR results (Fig. 5C) showed invalid detection for *23S rRNA* in 34 cases. Among 243 subjects included, 168 (69.1%, 168/243) had mutant-type *23S rRNA*, and 75 (30.9%, 75/243) had wild type. For *gyrA*, invalid detection occurred in 43 cases. Among 234 subjects included, 151 (64.5%, 151/234) had mutant-type *gyrA*, and 83 (35.5%, 83/234) had wild type. Both *23S rRNA* and *gyrA* mutations were present in 118 cases (50.9%, 118/232). *23S rRNA* mutation with *gyrA* wild type was found in 45 cases (19.4%, 45/232), *23S rRNA* wild type with *gyrA* mutation in 32 cases (13.8%, 32/232), and both wild type in 37 cases (15.9%, 37/232) (Fig. 5D).

### Correlation analysis between PCR-detected resistance genotypes and clinical parameters

**Fig 6:**
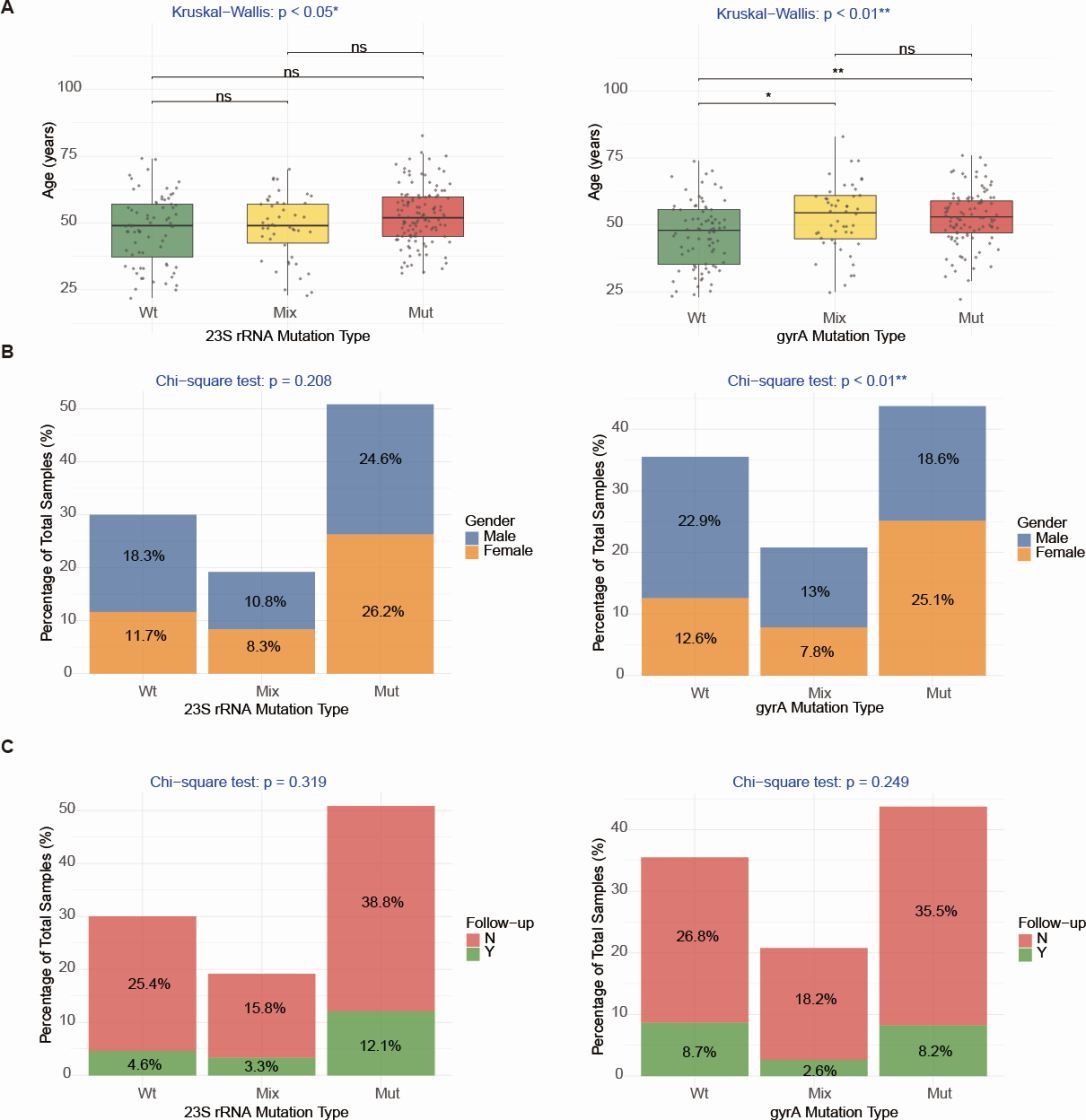
Correlation between PCR-detected resistance genotypes and clinical parameters. (**A**) Stacked bar chart relating *23S rRNA* and *gyrA* mutation status to age groups. (**B**) Stacked bar chart showing genotype distribution by sex. (**C**) Stacked bar chart comparing mutation status across follow-up categories. Follow-up No, First visit; Follow-up Yes, Follow-up visit. ^*^P < 0.05; ^**^P < 0.01; ns, significant.

Fluorescence quantitative PCR results (Table 3) indicated no significant differences in clarithromycin (χ² = 2.406, *P* = 0.121) or quinolone (χ² = 3.367, *P* = 0.057) genotype resistance rates between males and females. Similarly, no significant differences were found between primary diagnosis and retreatment groups for clarithromycin (χ² = 1.771, *P* = 0.183) or quinolone (χ² = 1.960, *P* = 0.161) genotype resistance rates. Compared to the <50-year group, the ≥50-year group showed no significant change in clarithromycin genotype resistance rate (χ² = 2.290, *P* = 0.130), but a significantly higher quinolone genotype resistance rate (χ² = 7.058, *P* = 0.008). Subjects were divided into three groups (wild type, mutant type, and mixed type) based on PCR results. Comparing the correlation between *23S rRNA* and *gyrA* genotypes and clinical parameters, it was found that subjects with mixed-type or mutant-type *gyrA* were significantly older than those with wild type (*P* < 0.05). Moreover, significant differences in *gyrA* mutation rates existed among different *gyrA* genotype groups (*P* < 0.01) (Fig. 6).

**TABLE 3:** qPCR analysis of resistance genotypes vs. clinical parameters^*a*^

Clinical parameters	*23S rRNA*				*gyrA*			
	Mut	Wt	*χ*²	*P* value	Mut	Wt	*χ*²	*P* value
Sex								
Male	85	46	2.406	0.121	75	52	3.367	0.057
Female	83	29			76	31		
Age (years)								
<50	72	40	2.29	0.130	60	48	7.058	0.008**
≥50	96	35			91	35		
Number of visits								
First visit	131	64	1.771	0.183	126	63	1.96	0.161
Follow-up visit	37	11			25	20		

^**^ *P* < 0.01. Mut, mutation; Wt, wild type.

### Consistency analysis between resistance genotypes and phenotypes for clarithromycin and quinolone

The overall concordance rates between Sanger sequencing results for clarithromycin and quinolone resistance genotypes and the corresponding phenotypes were 90.1% (95% CI, 85.2%–95.3%) and 93.3% (95% CI, 88.7%–96.1%), with Kappa values of 0.751 and 0.849, respectively. For fluorescence quantitative PCR, the overall concordance rates were 88.2% (95% CI, 83.9%–92.6%) and 92.6% (95% CI, 89.0%–96.2%), with Kappa values of 0.706 and 0.834, respectively (Tables 4 and 5).

**TABLE 4:** Agreement of clarithromycin resistance genotype with phenotype^*a*^

Assay method		E-test		CR % (95% CI)	Kappa value	*P* value
		Res	Sen			
Sanger seq	pos	137	6	90.1 (85.2–95.3)	0.751	0.000***
	neg	14	45			
qPCR	pos	141	7	88.2 (83.9–92.6)	0.706	0.000***
	neg	18	46			

^***^ *P *< 0.001. pos, positive; neg, negative; Res, resistant; Sen, sensitive; CR, concordance rate.

**TABLE 5:** Agreement of levofloxacin resistance genotype with phenotype^*a*^

Assay method		E-test		CR % (95% CI)	Kappa value	*P* value
		Res	Sen			
Sanger seq	pos	115	3	93.3 (88.7–96.1)	0.849	0.000***
	neg	9	53			
qPCR	pos	128	5	92.6 (89.0–96.2)	0.834	0.000***
	neg	10	60			

^***^ *P *< 0.001. pos, positive; neg, negative; Res, resistant; Sen, sensitive; CR, concordance rate.

## DISCUSSION

Recent multicenter investigations from China, across Asia, and in other world regions have similarly documented high and increasing *H. pylori* resistance rates (28, 29). The rising prevalence of antimicrobial resistance (AST) in *H. pylori* poses a significant challenge to its eradication. International guidelines discourage empirical antibiotic therapy in regions with high resistance rates. In this study, 277 *H. pylori*-positive individuals were enrolled. Based on antimicrobial susceptibility testing (AST) of gastric mucosal cultures, the phenotypic resistance rates to clarithromycin and quinolones were 73.8% and 67.7%, respectively. Sanger sequencing revealed genotypic resistance rates of 70.1% and 65.2%, while PCR indicated rates of 69.1% and 64.5% for clarithromycin and quinolones, respectively. All these rates exceed both the estimated rates in the literature (13) and the consensus threshold of 15%, defining a region as having high resistance. Thus, our findings confirm that the studied population belongs to an area of high phenotypic and genotypic resistance to both clarithromycin and quinolones. All three detection methods consistently showed that dual resistance to clarithromycin and quinolones was the most common pattern. This highlights the importance of basing antibiotic selection, especially when resistance to one agent is suspected, on AST results rather than empirical choice. Studies have confirmed that in high-resistance settings, AST-guided therapy achieves significantly higher eradication rates than empirical therapy (30). However, AST requires invasive collection of gastric mucosa and expertise in *H. pylori* culture. Therefore, sequencing and PCR for detecting resistance genotypes serve as crucial supplementary tools for clinical application.

In recent years, the use of stool samples combined with PCR for detecting resistance gene mutations has garnered considerable attention. Giorgio et al. (31) demonstrated concordance between stool and tissue PCR results in a small sample, but tissue PCR itself is not a consensus-recommended reference method, limiting the strength of this validation. Beckman et al. (32) validated PCR feasibility using a composite reference standard (breath test, endoscopy, culture, and urease test), but their study focused solely on clarithromycin resistance. Brennam et al. (33) compared quinolone resistance (*gyrA*) mutations in stool versus gastric tissue, finding a higher detection rate in stool. In our study, stool PCR detection rates were slightly lower than those from tissue culture, yet showed high concordance (92.3%) with tissue-based results, indicating promising application prospects. In summary, our study demonstrates that stool samples can be used to detect clarithromycin and quinolone resistance genotypes, with results showing good agreement with AST from gastric mucosa. This confirms the feasibility of using stool-based testing to assess clarithromycin susceptibility and provides important supplementary clinical data for stool-based PCR detection of quinolone resistance.

Furthermore, we analyzed key clinical parameters including gender, age, and treatment history. Neither phenotypic nor genotypic resistance was associated with gender or treatment history (primary vs. retreatment). Notably, both phenotypic resistance to clarithromycin and quinolones, as well as genotypic resistance to quinolones, increased significantly with age. This is likely attributable to the cumulative probability of exposure to these antibiotics over a lifetime. The clarithromycin resistance rates (both phenotypic and genotypic) in our cohort, including primary-treatment patients, were significantly higher than those reported in consensus guidelines and other studies (10). This may be attributed to the use of non-standard therapies and antibiotic overuse. While quinolone resistance rates in primary-treatment patients were comparable to earlier regional studies, the rates in retreatment patients were relatively low (34), suggesting a need for validation in multi-regional populations.

Sequencing identifies target gene point mutations to predict resistance. Our study found that A2143G and N87K were the predominant mutation sites in the *23S rRNA* and *gyrA* genes, respectively, consistent with previous reports (35). Mutations primarily involved single sites, though a few patients had two-site mutations in *23S rRNA*, and a minority had more than two mutation sites in *gyrA*. While the HelicoDR assay has been reported for combined detection of *23S rRNA* and *gyrA* mutations (27), it is not based on real-time PCR and is relatively tedious. Konrad Egli et al. (36) developed a real-time PCR method for detecting mutations in these genes, but it required gastric tissue samples, failing to meet the need for a non-invasive test. Compared to previous studies, our stool-based genotyping method offers the advantages of being non-invasive, convenient, and time-efficient while maintaining high concordance with phenotypic results. Therefore, stool-based genotypic detection holds promise as an alternative for assessing clarithromycin and quinolone resistance.

### Conclusion

In summary, clarithromycin and quinolone resistance rates are at high levels in this region and increase with age. Furthermore, the detection of clarithromycin and quinolone resistance genotypes demonstrates high concordance with phenotypic resistance results. Therefore, stool-based detection of these resistance genotypes can provide valuable guidance for selecting clinical treatment strategies for *H. pylori*-infected individuals in this region.

## SUPPLEMENTAL MATERIAL

Tables S1 and S2 spectrum.04062-25-s0001.xlsx Clarithromycin and fluoroquinolone resistance detection results of all samples by E-test, sequencing and PCR.

ASM does not own the copyrights to Supplemental Material that may be linked to, or accessed through, an article. The authors have granted ASM a non-exclusive, world-wide license to publish the Supplemental Material files. Please contact the corresponding author directly for reuse.
